# Case Report: A Case of Infant Bronchial Dieulafoy's Disease and Article Review

**DOI:** 10.3389/fped.2021.674509

**Published:** 2021-09-15

**Authors:** Yang Chen, Yiting Mao, Xingfeng Cheng, Ruihua Xiong, Ying Lan, Feng Chen, Furong Zhang, Jun Liu, Yanting Wu

**Affiliations:** ^1^Wuhan Children's Hospital (Wuhan Maternal and Child Healthcare Hospital), Tongji Medical College, Huazhong University of Science & Technology, Wuhan, China; ^2^The International Peace Maternity and Child Health Hospital, School of Medicine, Shanghai Jiao Tong University, Shanghai, China; ^3^Obstetrics and Gynecology Hospital of Fudan University, Shanghai, China

**Keywords:** bronchial dieulafoy's disease, critical care, rare disease, pediatrics, pulmonary

## Abstract

**Background:** Bronchial Dieulafoy's disease (BDD), characterized by constant diameter arterial malformation, is rare, especially among infants. The pathogenesis and clinical features of pediatric patients are unknown. Misdiagnosis and biopsy operations may lead to potential massive hemorrhage, which endangers the patient's life.

**Case Presentation:** Here, we present a case of a 9-month-old boy who was diagnosed with BDD with massive hemoptysis. The boy was cured by embolization of the bronchial artery and was in good health at the 1-year follow-up. In addition, we searched PubMed, Google Scholar, and Web of Science databases using keyword “Bronchial Dieulafoy's Disease (BDD)” and found six additional cases of pediatric BDD.

**Conclusion:** It is still insufficient to draw a conclusion about the origin of the disease. Bronchial angiography and endobronchial ultrasonography are considered promising methods to diagnose Dieulafoy's disease of the bronchus. Bronchoscopy with transbronchial biopsy should not be deployed due to the high risk of fatal hemorrhage. Explicit clinical case reports of BDD are needed to enhance the understanding of this rare disease.

## Introduction

**Figure 1:**
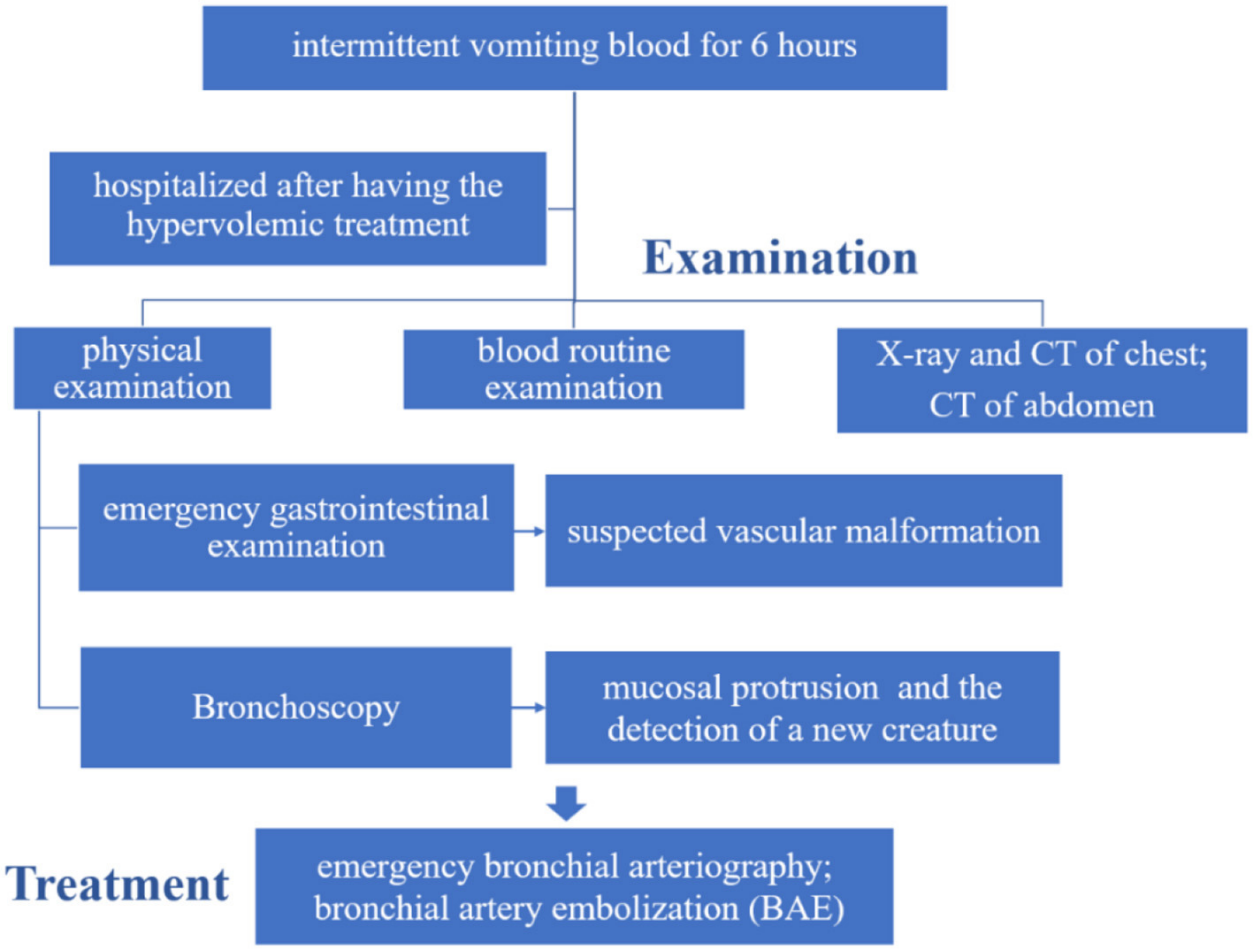
Timeline of the treatment process of the patient.

Dieulafoy's disease is a constant-diameter arterial malformation. It was first discovered in the digestive system in France in 1897 by Doctor Dieulafoy (1). Dieulafoy's disease in the digestive system is quite common. However, Dieulafoy's disease in the bronchi was first reported in 1995 by Sweerts (2) and has a relatively low incidence. At present, the pathogenesis of the disease is unknown. Most adult patients were reported having a history of smoking, chronic inflammation, or tuberculosis (3). The cases of infants are rarely reported. It is not clear whether there are congenital vascular malformations in infants and young children. Gastrointestinal bleeding and pulmonary hemorrhage are the major fatal symptoms of the disease. Due to insufficiency in the pathogenesis and clinical features of pediatric patients, misdiagnosis and biopsy operations of the lesion can cause fatal hemorrhage. Thus, warning and early recognition of the disease is crucial. Laboratory tests, bronchoscopy, computed tomography imaging, electronic gastroscopy, and gastric antrum pathology are fully described in this report. The aim of this report was to depict the clinical characteristics and the treatment process by representing this 9-month-old boy's case (Figure 1). Meanwhile, reviewing the clinical features of pediatric patients aged up to 14 years with BDD has great importance and clinical implications.

## Case Report

A 9-month and 3-day-old male infant with a chief complaint of “intermittent vomiting blood for 6 h” was admitted to Wuhan Children's Hospital on February 8, 2019. Without obvious incentives, the child presented hemoptysis after coughing with bright red blood a total of four to five times. Three of them were quite serious, and blood clots could be observed. No fever, rash, hematuria, or syncope was found. The boy was treated with anti-infectious drugs, hemostasis, and fluid supplementation at clinics. Then, he was transferred to our hospital for further treatment. He was hospitalized after hypervolemic treatment in the emergency room. The boy had been in good health, with regular vaccinations and no history of exposure to infectious diseases.

On physical examination, the boy's heart rate was 160 beats per minute, blood pressure was 97/34 mmHg, and respiratory rate was 35 breaths per minute. He was conscious but pale in color, with reduced skin elasticity. There were no bleeding points throughout the body, and the capillary filling time was 2 s. The superficial lymph nodes throughout the body were not swollen and enlarged, and the sclera had no yellow staining. The pupils had normal reflection of light. There was no congestion in the pharynx. The bilateral breathing movements were symmetrical. The lungs were clear. His heart rate was regular and strong, without a murmur. His abdomen was tender. A hemangioma was detected in the abdominal wall, ~1.5 × 1.5 cm in size, and there was no organomegaly. There were no abnormalities in the extremities and no restrictions on movement.

**Figure 2:**
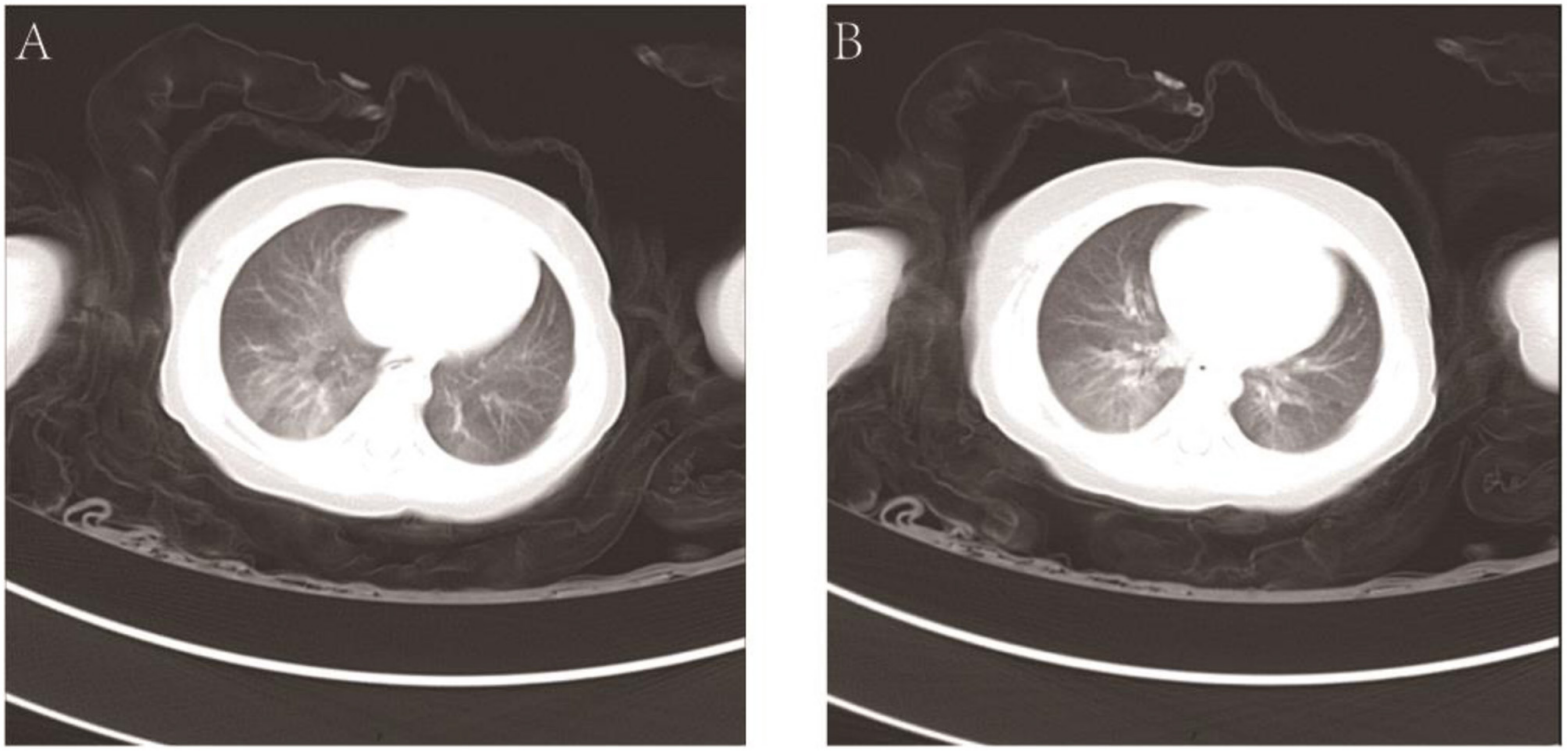
CT coronal scan of the lung **(A,B)**.

The hemoglobin level was 104 g/L, the hematocrit was 31.6%, the white-cell count was 7.70 × 10^9^/L, and the platelet count was 177 × 10^9^/L. The results of the coagulation test were as follows: the prothrombin time (PT) was 13.5 s, the activated partial thromboplastin time (APTT) was 28.8 s, the thrombin time (TT) was 22.1 s, and the fibrinogen (FIB) level was 1.02 g/L. Liver function tests showed that the total protein (TP) level was 43.5 g/L, the albumin (ALB) level was 26.4 g/L, the total bilirubin (TBIL) level was 3.8 μmol/L, alanine transaminase (ALT) was 31 U/L, and aspartate aminotransferase (AST) was 22 U/L. The X-ray slide of the chest noted bronchitis. Computerized tomography (CT) of the abdomen detected an abnormal density shadow on the left abdominal wall, indicating the possibility of hemangioma. Chest CT showed poor texture, uneven transmission, and ground-glass opacification (GGO) of both lungs. The GGO was more significant in the right lower lobe. Using the CT coronal scan to simulate bronchoscopy, no obstruction signs in the larynx, trachea, or main bronchi were found (Figure 2).

**Figure 3:**
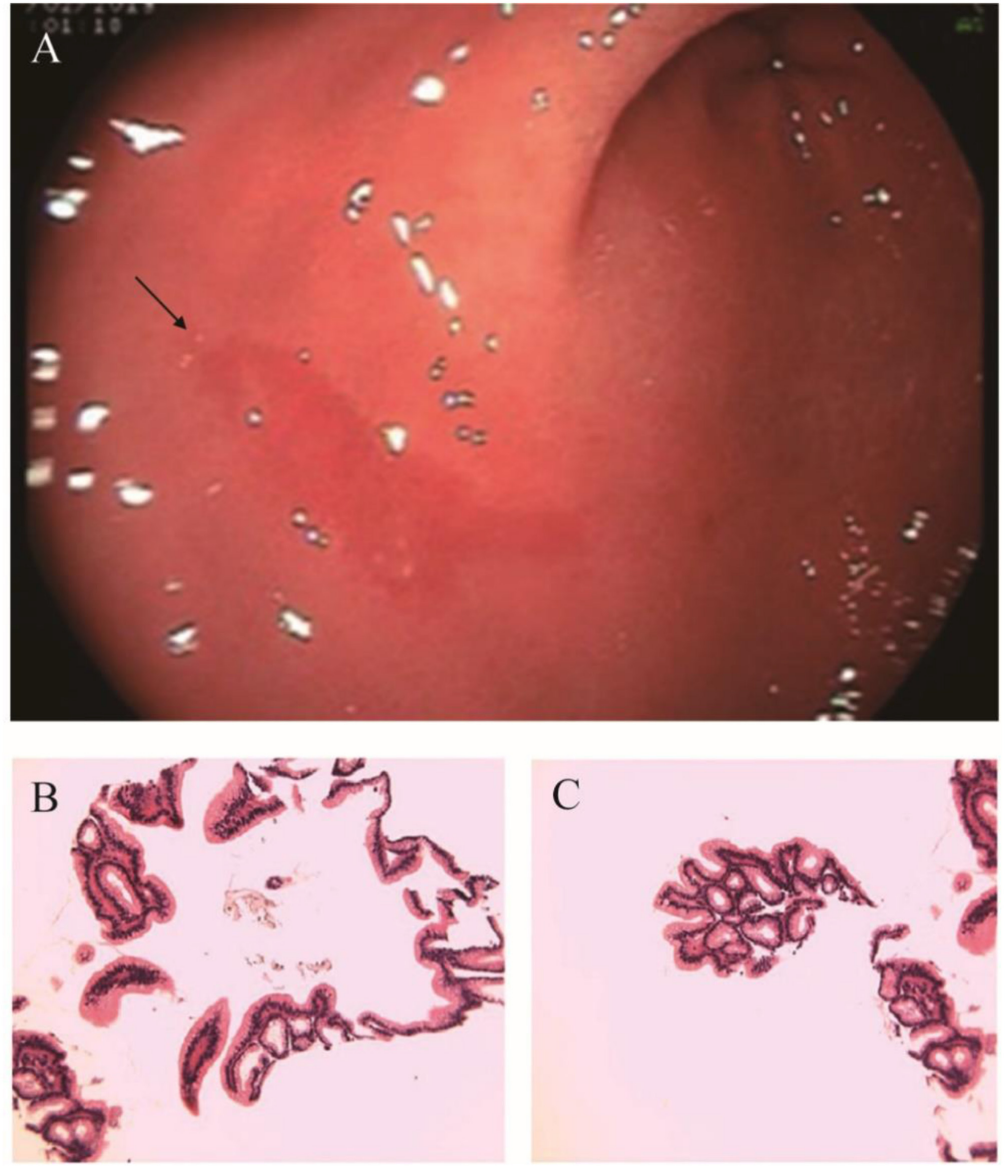
**(A)** Electronic gastroscopy. **(B,C)** Gastric antrum pathology.

**Figure 4:**
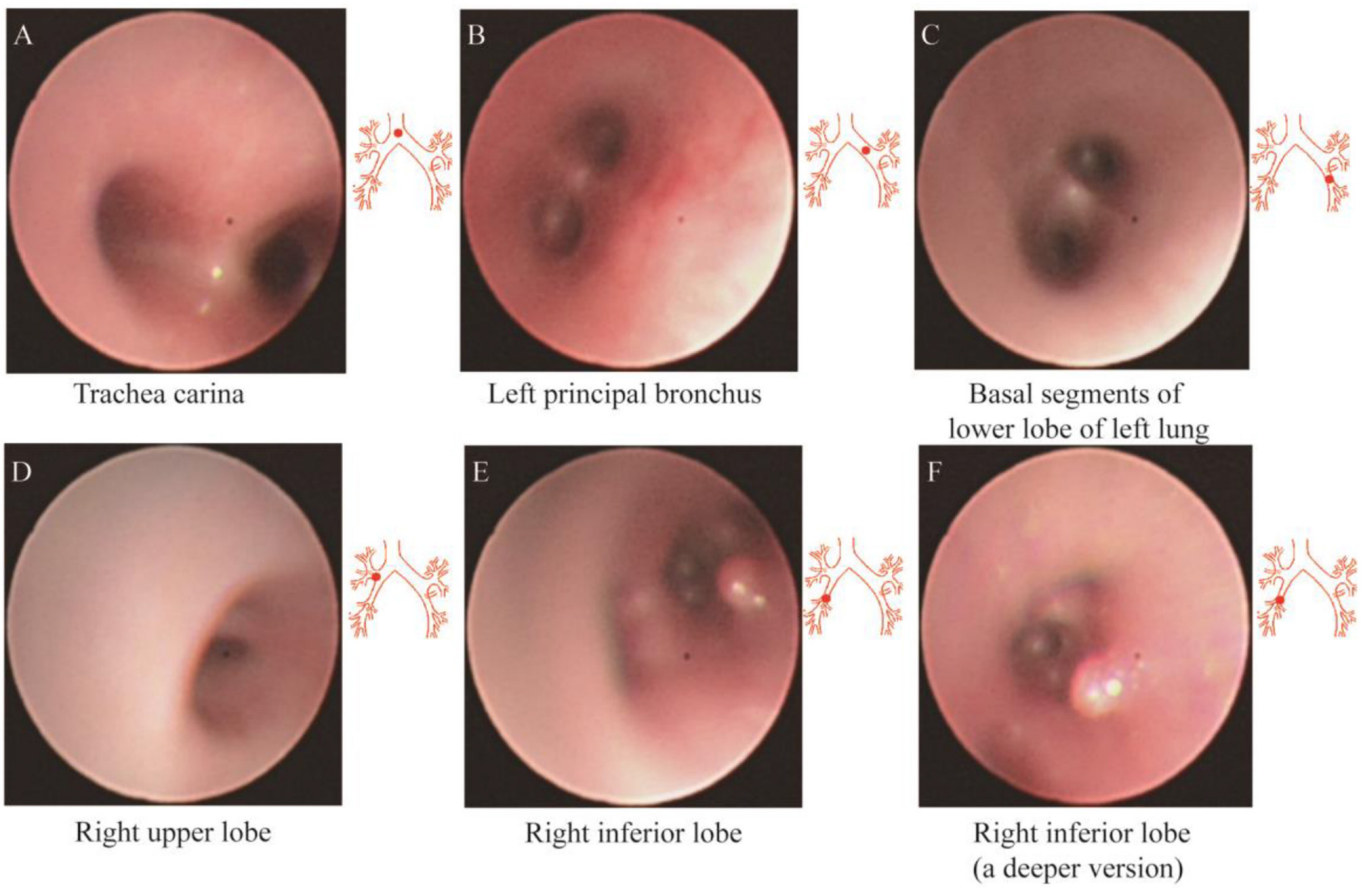
Fibrobronchoscopy of the left **(A–C)** and right **(D–F)** lobes of the bronchus.

After admission, the infant did not exhibit hematemesis again, although the coughing was still continuous. Black stool was observed once. Methods, such as hemostasis, acid suppression, and other symptomatic treatments, were received. An emergency gastrointestinal examination (Figure 3A) showed that there was patchy erythema near the corner of the stomach with a smooth surface and suspected vascular malformation. A biopsy was taken from the surrounding area. The pathology identified mild chronic inflammation of the mucosal tissue (Figures 3B,C). By conducting a thorough bronchoscopy (Figures 4A–F), we found a smooth mucosal protrusion in the opening of the right lower lobe. It was suspected of being a new formation, and bleeding after contact was obvious (Figure 4F). After spraying epinephrine and thrombin, the bronchoscope was removed until no more bleeding was observed.

**Figure 5:**
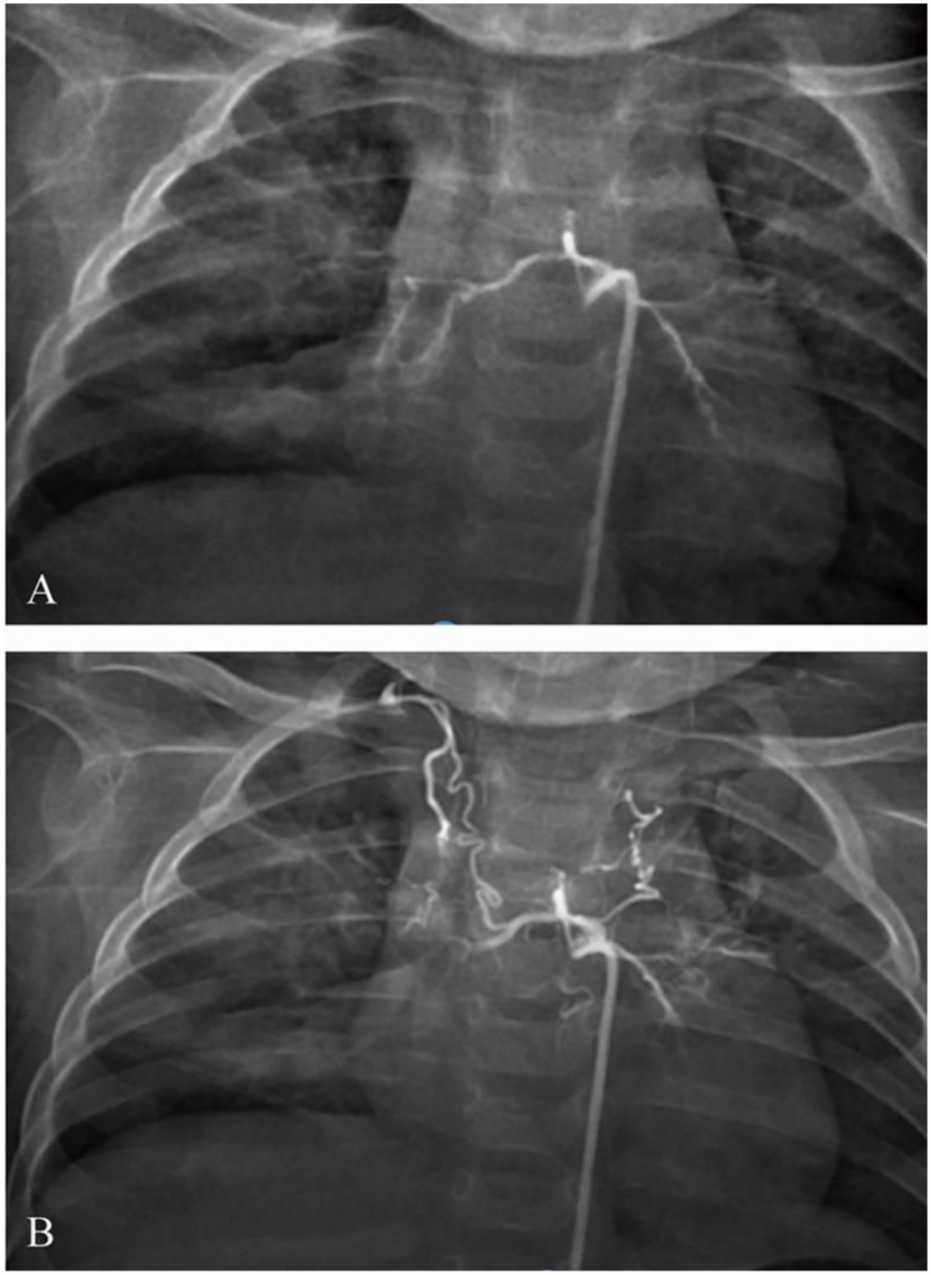
Bronchial angiography **(A)** before and **(B)** after embolism.

After bronchoscopy, the infant was transferred to the intensive care unit. The blood test was rechecked. The hemoglobin level was 70 g/L, the hematocrit was 22.6%, the white cell count was 5.79 × 10^9^/L, and the platelet count was 189 × 10^9^/L. Hemoptysis diminished, and the stool turned yellow. After 5 days, he developed recurrent hemoptysis three times after coughing, with ~20–30 ml of blood each time, accompanied by cyanotic lips and shortness of breath, ~60 times per minute. Emergency tracheal intubation, mechanical ventilation, volume expansion, and hemostasis were given. Urgent blood analysis showed that the hemoglobin level was 81 g/L, the hematocrit was 25.6%, the white cell count was 8.06 × 10^9^/L, and the platelet count was 315 × 10^9^/L. Therefore, under general anesthesia, the right femoral artery was punctured using the Seldinger technique, and a 5F Cobra catheter and a microcatheter were superselectively inserted into the bronchial artery. The angiogram showed that the bilateral bronchial arteries showed distortion; the distal end was disordered, blurred, and irregularly stained; and more contrast agents showed signs of spillage (Figure 5A). The 5F Cobra catheter was placed in the opening of the bilateral bronchial arteries; the microcatheter was inserted superselectively to avoid the intercostal artery; PVA (300 μm) and gelatin sponge particles (560–710 μm) + dexamethasone (5 mg) + contrast mixed embolization agent were used for arterial embolization via microcatheter; and the embolization was stopped when the contrast agent slowed down significantly. Re-examination angiography showed satisfactory embolization of the bilateral bronchial arteries (Figure 5B). Bronchoscopy was performed again after the operation. The new formation at the opening of the right lower lobe showed no significant bleeding. After 3 days, the trachea intubation was removed. The infant was discharged after the chest radiograph was found to be normal.

**Figure 6:**
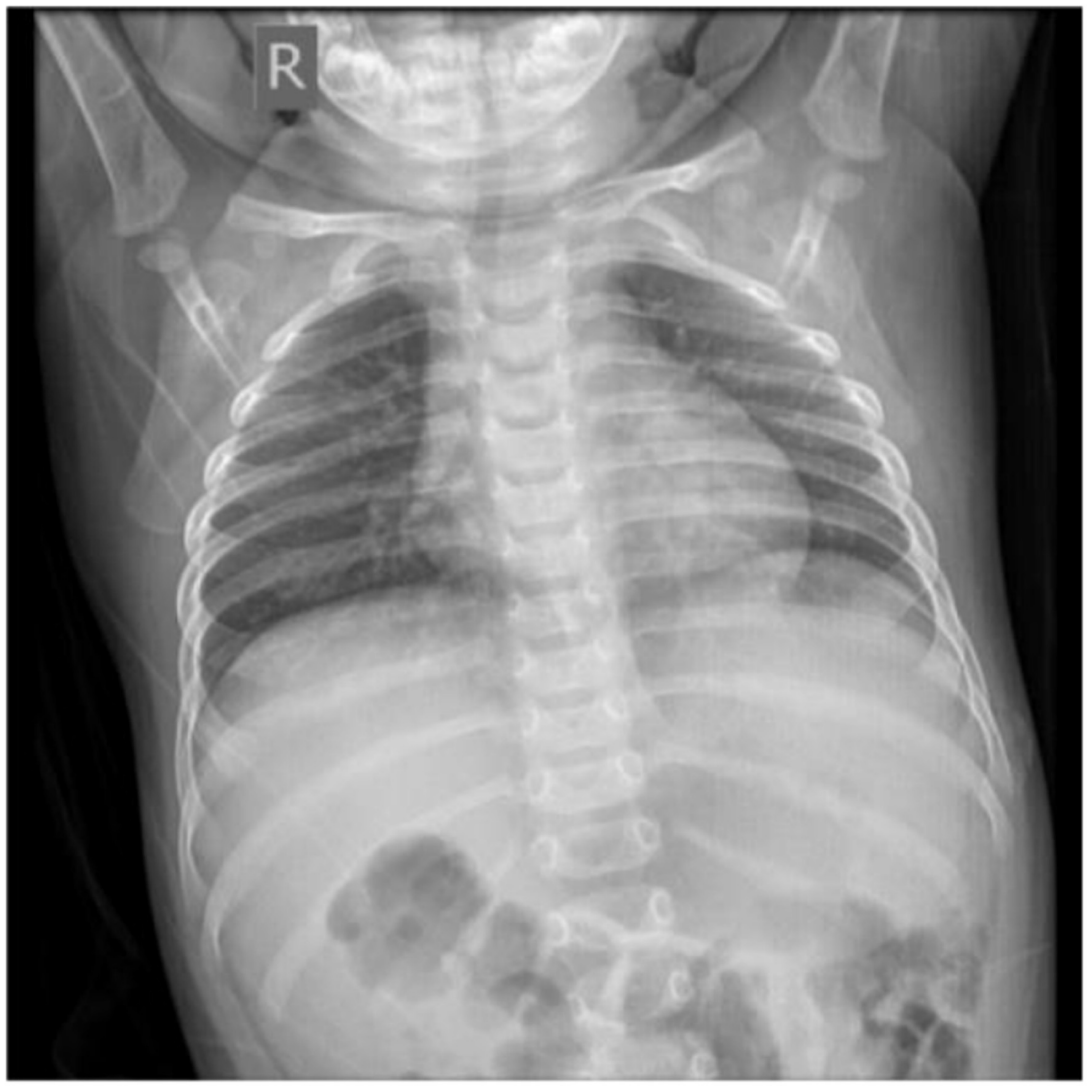
Chest X-ray after recovery.

Twenty-two days later, the chest X-ray slide of the infant showed no abnormal signs (Figure 6). We further followed up the current status of the infant 1 year later. He was in good health without coughing, pneumonia, hemoptysis, or vomiting blood. He showed no wheezing or discomfort after activities. The boy is now 1 year and 10 months old, with a height of 84 cm and a weight of 12 kg.

## Article Review

We searched PubMed, Google Scholar, and Web of Science databases by the key word “Bronchial,” “Dieulafoy's Disease,” and “pediatrics.” The aim of this study was to determine the clinical features of BDD pediatric patients aged up to 14 years. Six previous cases were reported. The following information was collected: pediatric patient characteristics, computerized tomography imaging, bronchoscopy, vascular angiography, and treatment. By analyzing the previous and present cases, the clinical characteristics are listed in Table 1.

**Table 1:** Characteristics of pediatric bronchial Dieulafoy's disease (BDD).

**References**	**Country**	**Sex**	**Age (years)**	**Respiratory history**	**Reason for admission**	**X-ray**	**CT**	**Bronchoscopy**	**Location**	**Biopsy and hemoptysis**	**Angiography**	**Source of vessel**	**Treatment**
Ganganah et al. (4)	Japan	Male	13	No	Intermittent hemoptysis over a week	NA	Ground glass opacity (GGO) of the right posterior basal lobe	Two elevated lesions with white caps	One at the entrance of the right lateral basal bronchus, one over the subcarina of the right lateral and posterior basal bronchus-black arrows	No biopsy	The right bronchial artery was tortuous and dilated, measuring ~2.7 mm in diameter	BA	BAE (recurred) + surgery (resection of the right middle and lower lobes)
Fang et al. (5)	China	Male	13	No	Massive hemoptysis	NA	Exudative lesion, consolidation, and atelectasis of right lower lobe	Two nonpulsating polypoid nodules of ~3–6 mm	Located at the carina between the external and posterior basal segment of the right lower lobe	No biopsy	A distal right branch of this bronchial artery was dilated and tortuous	BA	Surgery
Giordano et al. (6)	Italy	Male	11	No	Chest discomfort and massive hemoptysis	NA	Diffuse “ground-glass” opacity at the lower lobe of the right lung	NA	At the lower lobe of the right lung	No biopsy	A dilated and tortuous right bronchial artery	BA	BAE
Woodhull et al. (7)	China	Male	8	No	A sudden episode of severe hematemesis	Partial collapse of the right middle and lower lobe	An abnormally dilated right bronchial artery with collapsed right middle and lower lobes, and partial consolidation of the contralateral left lower lobe	Carina, left lower lobe, and lingula revealed minimal fresh and altered blood	At the right lower lobe	No biopsy	NA	BA	BAE
Yeh et al. (8)	China	Female	7	No	Frequent episodes of unprovoked massive hemoptysis for 5 months	NA	An abnormal vascular lesion in her bronchus intermedius	Patchy chronic inflammation in the submucosa and patchy hypertrophy of the subepithelial connective tissue	A polypoid lesion in right bronchus intermedius	No biopsy	Multiple tortuous enlarged feeding vessels	BA	BAE + surgery (a sleeve resection of the bronchus intermedius)
Niu et al. (9)	China	Female	8 months	No	Hemoptysis	NA	Flaky blur of the right upper lobe	Rough and erosive endometrium, bleeding	Upper lobe and middle bronchi of the right lung	No biopsy	Tortuous and dilated, beaded bronchial artery	BA	BAE (failed) + surgery (resection of the right upper lobe)
Chen et al. (this study)	China	Male	9 months	No	Intermittent hemoptysis	Bronchitis	Ground glass opacity of both lungs	A smooth mucosal protrusion	Opening of the right lower lobe	No biopsy	Distorted bilateral bronchial arteries	BA	BAE

## Discussion and Conclusions

Dieulafoy's disease, also known as constant-diameter arterial malformation, was first discovered in the digestive system in France in 1897 (1). Dieulafoy's disease in the bronchi was first reported in 1995 by Sweerts (2) and has a relatively low incidence. Recently, a review published in 2019 fully analyzed 73 cases of bronchial Dieulafoy's disease (BDD) (10). The youngest and oldest patients were 8 months and 85 years old, respectively, with an average age of 47.2 years. Dieulafoy's disease in the bronchi has a higher incidence in Asia. It has a greater occurrence in the right bronchus (~2/3). In the present case, the patient was admitted to the hospital due to symptoms of vomiting blood, although no obvious bleeding was found in the digestive tract. Thus, checking the presence of pulmonary hemorrhage by CT imaging of the lung is necessary. Meanwhile, dynamic observation of the gastroscopic performance of the gastrointestinal tract of the child is needed to evaluate the possibility of gastric Dieulafoy's disease.

By reviewing the pediatric Dieulafoy's disease, we found that these characters are also the same as discussed above. In the currently reported seven cases, four of them were hospitalized because of the massive hemoptysis, which indicates that the occurrence of the disease is rather dangerous. The source of malformation vessel was all bronchial vessel. The location of the lesions were all in the right lobe and can be cured by timely detection. The lesions were focal and single and rarely appear in the two lungs. Currently, bronchial artery embolism (BAE) is considered the first step of treatment and has been proven to be of great efficiency (11). Bronchial artery radiographic examination combined with embolization treatment plays a key role in the prognosis of stable patients or patients who cannot tolerate surgery. Three of them were in good health after BAE. Others chose to have lobe resection surgery due to the failure of BAE or recurrence of the disease.

Due to the lack of a clear chief complaint, misdiagnosis of this kind of patient is quite usual among infants. Our case was the second reported infant BDD. Two infant Dieulafoy's disease all occurred in China, and none of them has respiratory disease before. They also share common characteristics in the symptom, abnormal lesion's location, and blur in lungs. For infant BDD cases, a congenital developmental abnormality may play a role in the pathogenesis. Whether BDD could be diagnosed by following information such as symptoms (hemoptysis), new organisms that are prone to bleeding (especially on the right) and pulmonary angiography with vascular disorders and other manifestations are worth further discussion. Although with continuous development of diagnostic technology, especially the application of endotracheal ultrasound scans (12) and narrow-band imaging (13), vascular malformations under mucosal protrusion of this disease can be observed. Nevertheless, infants cannot undergo such tests due to their own limitations. Along with the previous cases (9) and this one, the disease is mainly diagnosed by bronchoscopy and angiography. As for all parents, they should be informed that hemoptysis or hematemesis is a serious symptom for children and needs careful medical care, follow-up, and auxiliary examination.

What is more, once bronchoscopy considers the possible diagnosis of BDD, biopsy should be performed extremely carefully. One case of adults BDD had biopsy in the first place, and the patient went through massive hemorrhage, which may lead to potential mortality (14). One case exemplified that even urgent endotracheal intubation, lung ventilation, and BAE were operated on after fatal hemorrhage caused by biopsy, and the patient ultimately died due to disseminative intravascular coagulation and multiple organ failure (15). Thus, more pediatric BDD patients are needed to fully analyze the pathogenesis, diagnosis, and medication of the disease.

Pediatric BDD is a disease with a relatively low prevalence. It is still insufficient to draw a conclusion about the origin of the disease. Bronchial angiography and endobronchial ultrasonography are considered promising methods to diagnose BDD. Bronchoscopy biopsy should not be deployed due to the high risk of fatal hemorrhage. Explicit clinical case reports of BDD are needed to enhance the understanding of this rare disease.

## Data Availability Statement

The original contributions presented in the study are included in the article/supplementary material, further inquiries can be directed to the corresponding authors.

## Ethics Statement

The studies involving human participants were reviewed and approved by Wuhan Children's Hospital (Wuhan Maternal and Child Healthcare Hospital), Tongji Medical College, Huazhong University of Science & Technology, Wuhan, China. Written informed consent to participate in this study was provided by the participants' legal guardian/next of kin. Written informed consent was obtained from the minor(s)' legal guardian/next of kin for the publication of any potentially identifiable images or data included in this article.

## Author Contributions

The study idea was conceived by Wuhan Children's Hospital, study design and analyses were developed by all authors. YC analyzed the patient data regarding the rare bronchial Dieulafoy's disease and the further treatment scheme. YM interpreted the material and was a major contributor in writing the manuscript. JL and YW revised the manuscript. All authors participated in the writing of the paper and critical discussions, read, and approved the final manuscript.

## Conflict of Interest

The authors declare that the research was conducted in the absence of any commercial or financial relationships that could be construed as a potential conflict of interest.

## Publisher's Note

All claims expressed in this article are solely those of the authors and do not necessarily represent those of their affiliated organizations, or those of the publisher, the editors and the reviewers. Any product that may be evaluated in this article, or claim that may be made by its manufacturer, is not guaranteed or endorsed by the publisher.

## Abbreviations

BDD: bronchial Dieulafoy's disease.
